# Combining Electrospray Mass Spectrometry (ESI-MS) and Computational Techniques in the Assessment of G-Quadruplex Ligands: A Hybrid Approach to Optimize Hit Discovery

**DOI:** 10.1021/acs.jmedchem.1c00962

**Published:** 2021-09-12

**Authors:** Giovanni Ribaudo, Alberto Ongaro, Erika Oselladore, Maurizio Memo, Alessandra Gianoncelli

**Affiliations:** Department of Molecular and Translational Medicine, University of Brescia, Viale Europa 11, 25123 Brescia, Italy

## Abstract

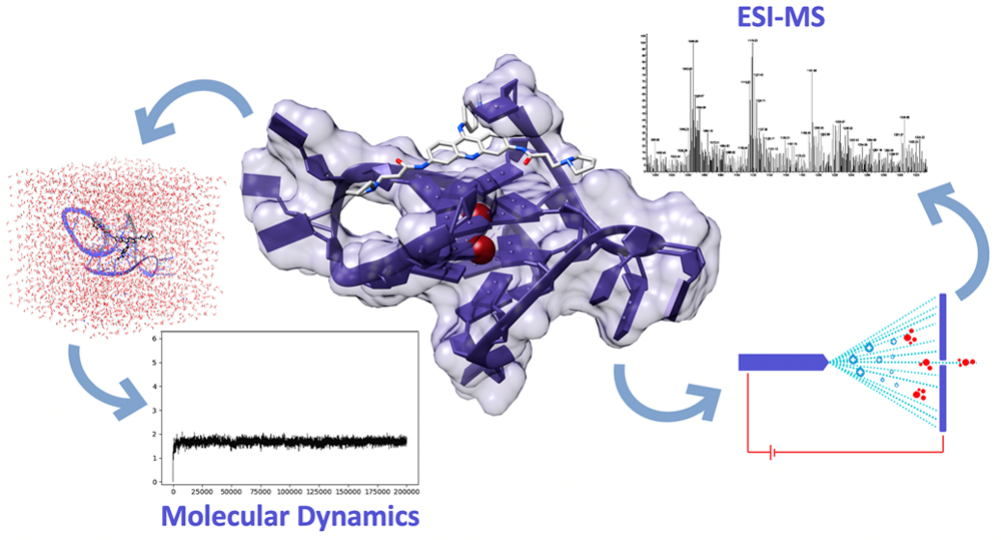

Guanine-rich sequences forming G-quadruplexes (GQs) are present in several genomes, ranging from viral to human. Given their peculiar localization, the induction of GQ formation or GQ stabilization with small molecules represents a strategy for interfering with crucial biological functions. Investigating the recognition event at the molecular level, with the aim of fully understanding the triggered pharmacological effects, is challenging. Native electrospray ionization mass spectrometry (ESI-MS) is being optimized to study these noncovalent assemblies. Quantitative parameters retrieved from ESI-MS studies, such as binding affinity, the equilibrium binding constant, and sequence selectivity, will be overviewed. Computational experiments supporting the ESI-MS investigation and boosting its efficiency in the search for GQ ligands will also be discussed with practical examples. The combination of ESI-MS and *in silico* techniques in a hybrid high-throughput-screening workflow represents a valuable tool for the medicinal chemist, providing data on the quantitative and structural aspects of ligand–GQ interactions.

## Introduction

Nucleic acids are flexible species that can fold into secondary structures consisting in a peculiar three-dimensional arrangement when in solution. This behavior is particularly favored by the formation of non-Watson–Crick hydrogen bonds patterns between nucleobases, which hold together the canonical double stranded DNA (dsDNA). Otherwise, when sequences containing guanines are bonded by a Hoogsten base pairing, they can generate a planar array with the shape of a tetrad. These arrangements stack one over the other and are further stabilized by positive ions interacting with the O6 lone-pair electrons of guanines.^1^ The formation of inter- and intramolecular structures of the resulting assemblies, known as G-quadruplexes (GQs), was confirmed both *in vitro* and *in vivo*.^2^ GQs can be constituted by both deoxyribonucleic acid (DNA) and ribonucleic acid (RNA).^3^

GQs indeed comprehend a wide family of diverse structures, as different strand polarities and the orientation of interconnecting loops define several topologies.^4,5^ In this connection, guanines can adopt a *syn*- or *anti*-orientation of the glycosidic bond, and the overall topology is defined as parallel, antiparallel, and hybrid. Such conformational aspects are described more in detail by Cang et al. in their contribution.^6^ Depending on these, the connecting loops are defined as diagonal, lateral, or double-strand reversal (propeller loop). The organization of such loops also influences the shape of the grooves, which are cavities bounded by phosphodiester backbones.^7^ Moreover, some GQ-forming sequences have been reported to be polymorphic. For example, human telomeric DNA, which is characterized by the presence of the TTAGGG repeat, can adopt an antiparallel GQ structure stabilized by sodium ions or a hybrid GQ in the presence of potassium.^8,9^

Previous studies described the presence of G-rich GQ-forming sequences in different genomes, ranging from viral to human.^3,10,11^ Computational predictions outlined that more than 700 000 GQ-forming DNA sequences can be retrieved in the human genome.^12,13^ Moreover, biological studies highlighted the prevalence of GQs at gene regulatory regions, in telomeres, in chromatin DNA, and in specific RNA sequences. As anticipated, such arrangements are generally characterized by both structural polymorphism, which also depends on the involved sequence, and flexibility.^7^

Since enzymatic machineries that process DNA or RNA are hindered by GQs, the combination of inducing GQ formation and stabilizing them with small molecules represents a strategy for interfering with key cellular functions such as transcription, translation, and telomerase activity.^3,14−16^ Beside the more widely studied field of GQ involvement in uncontrolled cellular proliferation and cancer progression, growing evidence suggests the relevance of GQ nucleic acids, and of RNA in particular, in neurons. In these cells, GQs influence the formation of stress granules, shedding new light on possible mechanisms to be targeted in neurodegenerative diseases.^17−20^ It must also be pointed out that GQ-forming sequences were recently identified in the genomes of several viruses^21^ and coronaviruses in particular, including severe acute respiratory syndrome-coronavirus-2 (SARS-CoV-2).^22,23^ Moreover, Zhao et al. reported that the stabilization of specific SARS-CoV-2 GQ RNA sequences with small molecules contrasted the translation of the N protein both *in vitro* and *in vivo*.^24^ Moreover, in the quest for novel strategies to contrast human immunodeficiency virus (HIV), a recent study demonstrated that a RNA GQ ligand can interfere with unfolding, DNA–RNA duplex formation, and subsequent reverse transcription.^25^

The interaction of drug candidates with GQ sequences is currently commonly studied by spectroscopic methods, which will be briefly outlined in the following. Nevertheless, determining the details of the interaction at the molecular level can be challenging when relying on conventional analytical techniques. The current paper will focus on the combination of two innovative and evolving methodologies used in the investigation of the interaction of small molecules with GQ arrangements. Electrospray ionization mass spectrometry (ESI-MS) is a flexible experimental analytical technique that is constantly being improved and optimized to study these noncovalent assemblies in the gas phase. As will be discussed in the following, nondenaturing “native” ESI-MS has emerged as a high-capacity drug discovery tool in this context. On the other hand, computational studies, particularly molecular modeling of ligand–GQ complexes, are perfect partners for setting up a highly efficient screening workflow. These two techniques have more in common than one can expect at first glance, from their theoretical basis to practical aspects, and their interplay represents an attractive strategy for building an efficient ligand-screening workflow. Current review articles do not generally cover the topic of such innovative techniques applied to the discovery of GQ ligands from the perspective of drug design. Previous comprehensive contributions on the use of ESI-MS as a GQ ligand screening tool date back to the previous decade^26−29^ or are specifically focused on a single technique and are limited to an audience of specialized readers.^30^ Moreover, the combination of ESI-MS with computational tools has only been briefly depicted in the context of ligand–GQ interactions.^31^ On the other hand, basic and more conventional experimental methods for studying such bindings have been more extensively discussed through the years.^32−34^

More than 160 scientific contributions in the literature in the time frame from 2000 to 2020 were considered and screened for the preparation of this paper. Original research articles were retrieved by searching the PubMed (www.ncbi.nlm.nih.gov/pubmed/) and Scopus (www.scopus.com) databases using keywords such as “G-quadruplex”, “RNA”, “DNA”, “mass spectrometry”, “ESI-MS”, “docking”, “molecular modeling”, and “molecular dynamics” as well as their combinations.

### G-Quadruplex Binders

The growing understanding of the GQ structure and function led to the design and development of low-molecular-weight ligands capable of interfering with these arrangements. Ideally, to trigger one of the biological effects cited above, a GQ ligand should modulate the stability of the structure even when in the presence of other potential targets such as dsDNA.^33^ According to the literature, the classification of such binders has generally operated on the basis of their chemical nature or in light of the preferential interaction motif with GQs. Such features are indeed interconnected; ligands bearing planar aromatic scaffolds generally stack on the quartets, while positively charged molecules interact with grooves and loops. Compounds characterized by the presence of a positive charge that binds to the center of a quartet may also stabilize the GQ.^7^ As anticipated anyway, the ligand must be characterized by a marked selectivity for GQ over dsDNA to act as an efficient GQ binder and stabilizer and to limit cross reactivity and side effects at the same time.^35,36^ Chaudhuri et al. recently published an updated and detailed report that overviews the evolution of small molecules interacting with GQs. In this contribution, the authors reported a rational classification of the compounds based on their chemical structures.^37^

The so-called π-stacking ligands were originally developed starting from dsDNA intercalating agents. These compounds generally possess a large aromatic scaffold and side chains that can be protonated, thus improving the water solubility and providing additional sites for binding grooves and central channel through electrostatic interactions. These molecules stack on the top of a tetrad, an event which is favored due to the low dissociation and destacking that make intercalation more unlikely.^7,38^ This is the interaction motif of BRACO-19,^39^ some nonpolycyclic aromatic ligands,^40^ several natural compounds^41−44^ and metal complexes.^45^

**Figure 1:**
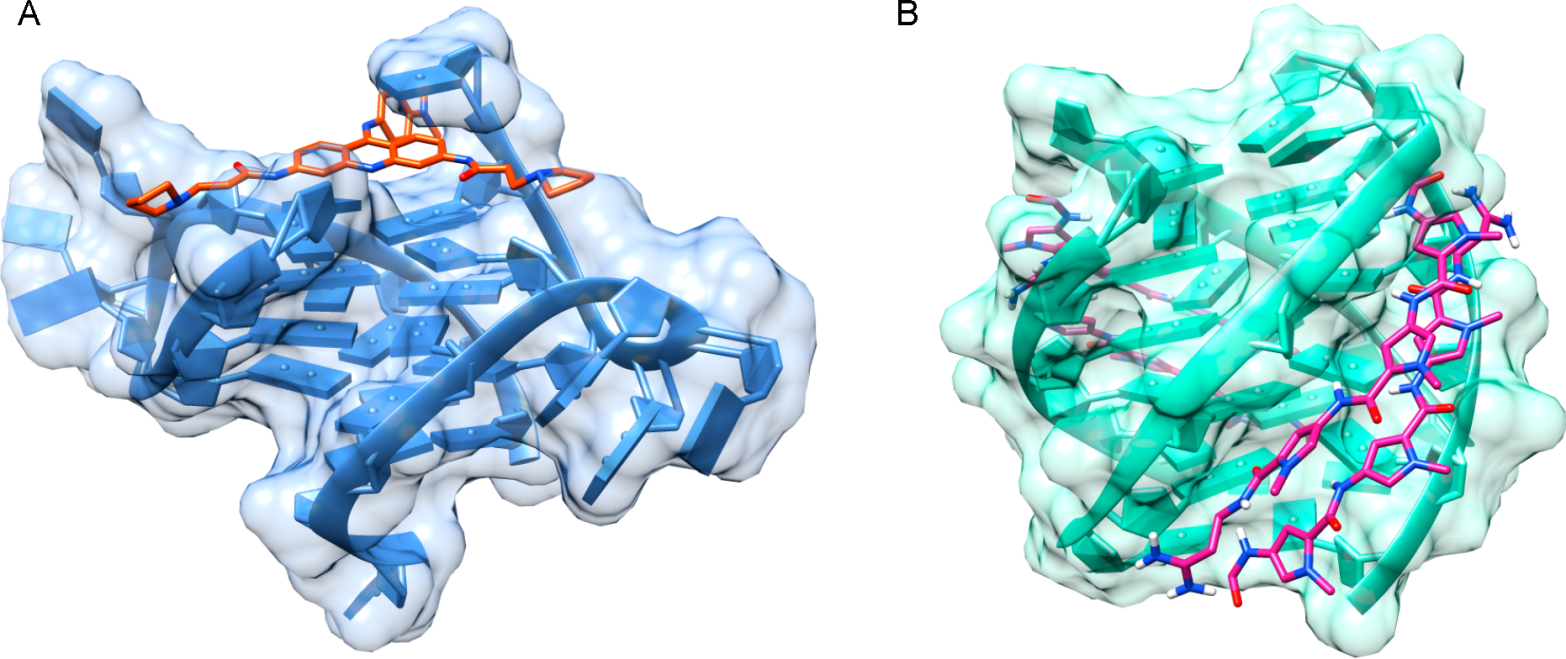
(A) BRACO-19 interacts with GQ via stacking (PDB ID 3CE5). (B) Distamycin is a GQ groove-binding agent interacting with GQ with a 2:1 stoichiometry (PDB ID 2JT7).^48^ Molecular graphics images were produced using the UCSF Chimera package from the Resource for Biocomputing, Visualization, and Informatics at the University of California, San Francisco, California (supported by NIH P41 RR-01081).

While π-stacking is the most common interaction motif observed for GQ binders, other patterns have also been reported. In more detail, groove- and loop-binding compounds take advantage of conformational differences between these sites in dsDNA and GQ. This is the interaction pattern reported for distamycin and its derivatives (Figure 1).^46^ Moreover, some planar compounds that may have been thought to interact by stacking were found to bind grooves and loops in light of the presence of a positive charge on the scaffold.^47^

A third peculiar pattern was reported for some molecules that target GQs by binding the central channel in combination with other interactions. This is the case of compounds bearing an anthracene moiety, which promotes stacking, that has a substitution in the 9 position with a polyamine chain that, when protonated, mimics the ions of the central channel.^49^

In their review, Yuan et al. presented an overview of the ligands capable of recognizing GQ nucleic acids. The authors classified the compounds in the following groups: organic ligands, inorganic ligands, and natural products.^29^ This alternative classification sheds light on the relevance of natural and nature-inspired ligands reported through the years as GQ binders or stabilizers.^50^ Telomestatin, a macrocyclic compound isolated from *Streptomyces anulatus*, is a widely studied example from this class as it is one of the most potent and selective GQ binders.^51^ Berberine^52,53^ and natural flavonoids, such as quercetin and rutin, represent other examples.^42,44^ On the side of small molecules prepared by organic synthesis, it must be noted how the *in situ* generation of GQ ligands is becoming a trending perspective in recent years. The use of innovative synthetic techniques, for example, those based on nanotemplates and click chemistry, expand the toolbox of the medicinal chemist in the quest for novel ligands.^54,55^

Besides preliminary *in vitro* studies, a considerable number of GQ ligands showed antiproliferative effects *in vivo*, even if some of the compounds that belong to the chemical classes described in this paragraph are traditionally endowed with uncertain adsorption, distribution, metabolism, excretion, and toxicity (ADMET) profiles and a limited drug-likeness.^34,39^ Nevertheless, a growing number of very recent contributions in the literature support the interest of G-quadruplexes as druggable targets and describe the potential application of ligands in the regulation of neural gene expression,^56^ in the interference with viral replication^57^ and as novel therapeutic agents against uncontrolled cellular proliferation.^58^ The reader is invited to refer to the paper by Spiegel et al. for a more comprehensive overview of the biological effects regulated by G-quadruplexes.^15^

### Methods and Techniques for Studying Ligand–GQ Interactions

Experimental techniques conventionally used to study ligand–GQ interactions range from simple methods to advanced experimental setups.^7,30^ Basic methods can be adopted to evaluate approximate ligand affinity, while more sophisticated techniques are employed to study thermodynamic, kinetic, and conformational properties of the interaction. Ideally, to provide support to the medicinal chemist in the search for GQ ligands, analytical techniques should unambiguously allow the measurement of the GQ over dsDNA selectivity and provide insights about binding sites and the mode of interaction.^59^ Nevertheless, a more detailed and focused discussion of such experimental techniques, the theoretical aspects, and the measured physicochemical properties does not fall within the scopes of the current paper. A brief overview will be provided anyway in the following to put the subsequent discussion in the right context, and the interested reader is invited to refer to other reviews that are more focused on biophysical techniques.^7,33,60,61^

Optical spectroscopy, comprehending UV–vis, fluorescence, and circular dichroism (CD) are well established and routinely adopted techniques to investigate ligand–target interactions. In combination with melting temperature measurements, stabilization effects can also be evaluated.^62^ Isothermal titration calorimetry (ITC) and surface plasmon resonance (SPR) are techniques that help to quantitatively determine the binding events, providing measurements of thermodynamic and kinetic parameters as well as the stoichiometry of the interaction.^63,64^ Investigations based on X-ray diffraction and nuclear magnetic resonance (NMR) spectroscopy are generally endowed with structural insights and are of fundamental relevance, considering their differences, for building the templates used for *in silico* studies that will be discussed in another section.^65,66^ Another category of assays is constituted by *in vitro* biochemical techniques, such as the telomere repeat amplification protocol (TRAP assay). This test measures the elongation of a telomeric strand in order to indirectly measure the effect of a ligand on a GQ sequence.^67^ Other tests, such as the Taq polymerase stop assay and the PCR stop assay, also allow an analysis of the effects of the binders in nontelomeric GQs.^68,69^

## Electrospray Ionization Mass Spectrometry

Increased instrument availability and improved flexibility pushed the development of ESI-MS as an always more commonly used tool for the discovery of GQ binders along with the traditional techniques cited above.^28,70^ In general, mass spectrometers operate via the separation of ions, which in the current case are made up of ionized and desolvated ligand–GQ complexes. This event occurs in high vacuum. While all types of mass analyzers can potentially be used for the investigation of ligand–GQ complexes, ESI has emerged as the ideal soft source in light of its features that will be discussed in the following.^28,71−73^ Nevertheless, some examples of MS-based studies describing the use of matrix-assisted laser desorption ionization (MALDI) sources were also reported.^74^ In particular, MALDI has found application in the investigation of the interaction between GQ sequences and protein assemblies.^75,76^ More generally, ESI is commonly used for the analysis of biomolecules such as peptides, proteins, and nucleic acids. Preliminary observations of intact dsDNA sequences date back to the 1990s, together with early ESI-MS reports on duplex-ligand interactions, and rapidly evolved into a screening tool suitable for drug discovery.^77^

In an ESI source, the solution containing the analyzed species flows through a capillary on which a high voltage is applied, promoting the vaporization of the sample solution in charged droplets. In this specific case, the sample is represented by an aqueous solution of the analyte that is infused at atmospheric pressure. The overall process could be described in three steps: droplet formation, droplet fission, and separation of desolvated ions.^28^ To a certain extent, in this specific context ESI acts by “extracting” biomolecules that are already ionic in solution, as nucleic acids are polyanions (p*K*_a_ < 1). Thus, they are preferably analyzed in the negative ionization mode, and a negative voltage is applied on the capillary. The entrance of the spectrometer is at ground, and the electric field promotes the movement of the ions and the emission of charged droplets, which contain an excess of ions of a certain polarity, from the tip of the capillary.^28^ The resulting negatively charged droplets progressively undergo fission into smaller droplets due to collisions with the ambient gas. As the droplet radius decreases, Coulomb repulsion increases until a critical radius is reached, called the Rayleigh limit, and an asymmetrical explosion of the particle occurs. Subsequent progressive fissions into even smaller droplets eventually provide isolated polyanionic species in shells of the remaining solvent and counterion molecules. The final step of this mechanism leads to the formation of desolvated ions in the gas phase; in the so-called “charge residue” model, a droplet contains a single molecule of the analyte ion, which in the current case is represented by a macromolecule and, more specifically, a nucleic acid sequence potentially complexed with a ligand.^28,60^ As will be discussed more in detail in the following, by tuning voltages, temperatures, and pressures in the spectrometer, the opportune removal of solvent and counterions, which are respectively termed desolvation and declustering, can be achieved to optimize the signal of the investigated species.^60,78^ In these conditions, minimal fragmentation occurs, and as a result noncovalent interactions are not altered during the ESI process.^28^

More specifically, native mass spectrometry conditions allow the transfer of an intact protein, nucleic acids, and macromolecular assemblies into the gas phase from a solution. Minimal disruption of the noncovalent interactions present in the solvated form must be achieved to efficiently study higher-order structures, folding, and noncovalent complexes.^30,79,80^

ESI-MS studies allow the determination of masses of single species, thus directly providing information on involved nucleic acid strands, bound cations, and the stoichiometry of the ligand complex.^33,81^ These insights are extremely useful and can be used, for example, to guide the researchers in choosing the right fitting models for other screening techniques, such as spectroscopic methods. MS is a technique that has the advantage of going beyond the apparent ligand binding affinity, as it helps to distinguish among different folding and binding equilibria separately.^82^ Moreover, if signals of different complexes are distinguishable and the resolution is appropriate, competition experiments can be carried out to highlight the most efficient ligand or the preferred target of a set to be further investigated by means of other methods.^60^

Previous studies aimed to investigate the reliability of ESI-MS ligand screening studies by comparing the measurements obtained by this technique with fluorescence melting data. Experiments performed on different GQ structures and via investigating different ligands were in good agreement.^83−85^ In particular, this technique has been reported to effectively avoid providing false positives.^86^ Additionally, for quantitative purposes ESI-MS analysis does not require any titration, and binding constants can be determined from a single spectrum. Nevertheless, calculated binding constants should be determined at different concentrations of the ligand since equilibrium binding constants should not be affected by this parameter.^28^ It must be also noted that an excess amount of ligand can be added to estimate the maximum binding stoichiometry.^60^

In summary, ESI-MS has emerged as an efficient screening technique for studying ligand–GQ complexes thanks to its features in terms of sensitivity and low sample consumption. This technique, as will be discussed in the following, can in fact potentially provide information on the binding mode, sequence selectivity, and the ligand binding affinity.^34^

### Tuning Instrumental Parameters and Experimental Conditions

Native ESI-MS should ideally allow the investigation of a GQ sequence in the spectrometer while preserving the arrangement present in solution, which is stabilized by monovalent cations. In this connection, the first experimental aspect that must be considered consists of preparing a nucleic acid solution of an opportune ionic strength, typically ranging from 100 to 150 mM. ESI-MS is generally characterized by a limited salt tolerance due to the counterion effect on nucleic acid molecules, which results in a wide population of adduct species in the presence of sodium. Thus, ammonium acetate is traditionally preferred as a sample preparation buffer as it limits the formation of adducts. In addition to this, GQ-forming sequences generally fold in a similar arrangement when complexing ammonium and potassium, which is a biologically relevant cation.^85^ Nevertheless, in some cases the formed structures can be sensitively different.^87^ In the negative ionization mode, polyanionic nucleic acid is accompanied by an excess of acetate ions. Proton transfer reactions from NH_4_^+^ to PO^–^ promote the neutralization of phosphates and, since ammonium acetate is in excess, only a fraction of the phosphates remain negatively charged. This mechanism justifies the generally observed charge states for oligomers (from −4 to −7 for a 23-mer GQ).^85,88,89^ Recent improvements of the experimental conditions allow the study of GQs in a condition that is both closer to the physiological environment and more significant from a biological point of view using potassium as the cation for sequence stabilization.^90^ The former approach consisted of folding the GQ in physiological potassium concentrations and the subsequent removal of the noncoordinated ions by filtration or ethanol precipitation.^70,91^ Another strategy is based on GQ folding in a low concentration of potassium (1 mM), thus not affecting the ionization, together with a volatile bulky buffer such as triethylammonium acetate. This ion does not fit inside the GQ so it does not compete with potassium, but it provides sufficient ionic strength for an efficient ionization. Scalabrin et al. recently reported an improved method based on the latter approach. The authors described a mixture of isopropanol/triethylamine/hexafluoroisopropanol as optimal solvent conditions and obtained a high sensitivity for GQ species in their ESI-MS studies (40 nM).^90^ In this context, another parameter that may influence GQ formation and stability is pH. By comparing ESI-MS spectra results from the analysis of the same sequence, it has been observed that the formation of GQ is favored at pH 4, while random-coil DNA was preferentially detected in alkaline conditions.^29,88^

Some spectrometers allow the direct acquisition of samples prepared in aqueous solutions, but 10–20% of an organic cosolvent (usually methanol or isopropanol) can be added to the sample to increase its volatility and, consequently, the signal-to-noise ratio.^60,90,92^ One of the most reliable procedures involves the addition of 15–20% methanol to the samples prior to infusion to decrease surface tension of the droplets. Moreover, it has been demonstrated by CD analysis that methanol addition does not promote conformational changes and variations in terms of peak ratios.^28^ On the other hand, higher concentrations of organic solvents (*e.g.*, 50% methanol) can promote structural alterations and trigger the conversion to a different GQ topology.^88,93,94^

Instrumental setup and conditions, as it generally happens in analytical chemistry, unavoidably influence the results of the experiment. Nevertheless, in the specific case of the GQ–ligand interaction analysis, the crucial step is represented by the efficient droplet formation and desolvation. On the other hand, an excessive amount of energy could promote the disruption of noncovalent interactions and, eventually, fragmentation. Therefore, source and capillary temperatures as well as acceleration voltages should be kept as low as possible, and ESI is by far the most flexible source that meets these requirements.^28,90^ Source “softness” is generally evaluated based on the presence, in the *m*/*z* spectrum, of few remaining ammonium ion adducts on the nucleic acid. Of course, in the context of GQs, if the resolution is sufficient these should be detected in the correct stoichiometry with respect to the number of quartets formed by the sequence (*n* – 1).^95,96^ The number of cation adducts is indeed indicative of the number of tetrads involved in GQ formation.^89,97^

**Figure 2:**
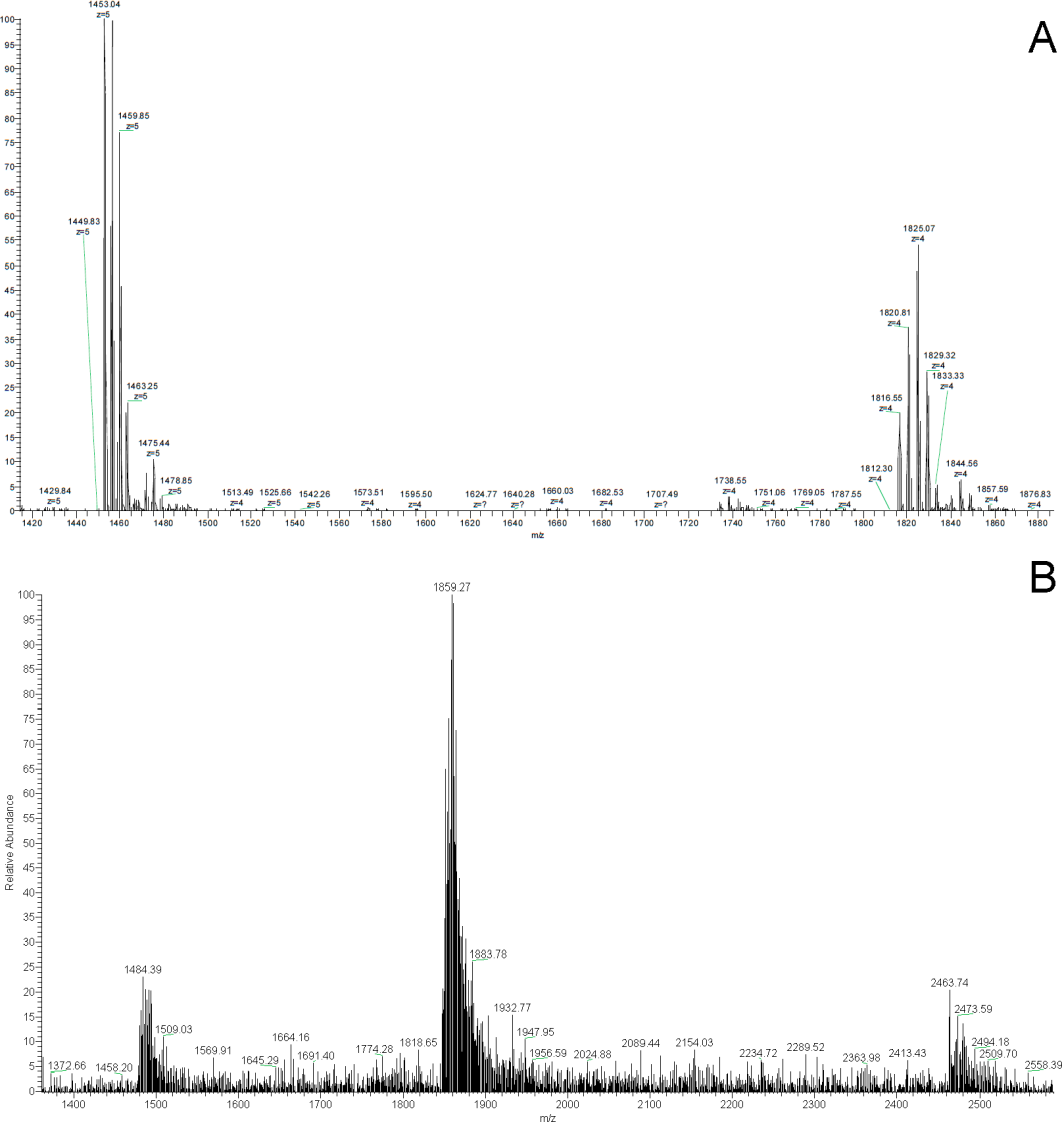
Comparison of the spectra resulting from the acquisition of the same sample (10 μM human telomeric GQ forming sequence 5′-AGGGTTAGGGTTAGGGTTAGGGT-3′ in 150 mM ammonium acetate, negative ionization mode) with (A) LTQ Orbitrap Velos and (B) LCQ fleet ion trap instruments.

Other instrumental performances, such as resolution and sensitivity, are related to the technology on which the mass analyzer is based. In previous studies from our group, the ligand–GQ interaction was studied using several ESI instruments based on different setups: a LCQ fleet ion trap (Thermo Fisher Scientific, Waltham, MA) instrument,^42,44^ a Xevo G2-XS QTof (Waters Corporation, Milford, MA) instrument,^98,99^ and a LTQ Orbitrap Velos (Thermo Fisher Scientific, Waltham, MA) instrument.^89^ A higher resolution allows the resolution of a more complex mixture of species. This is desirable, particularly when carrying out a competition assay between compounds generating ligand–GQ complexes with similar molecular weights. In fact, it is not uncommon to observe overlaps between signals at higher charge states.^100^ More in general, high-resolution instruments facilitate the assignment of the charge of a peak from the isotopic distribution (Figure 2).^28^

Concerning sensitivity, it must be noted that ESI-MS is a very sensitive technique *per se*, especially when compared to more traditional spectroscopic methods used to probe ligand–GQ interactions, and sample volume requirements are low. With a typical experimental setup, a 20–100 μL sample at a nucleic acid concentration of 1–10 μM is sufficient for routine acquisitions.^28,44^ Of course, this is absolutely indicative, as the required sample volume varies basing on the injection system. Nevertheless, it can be stated that ESI-MS generally operates in the range of picomole concentrations. Concerning the flow rate, when measurements are carried out with a conventional ESI source, a 0.1–5 μL/min rate can be used.^42,89^

### Quantitative Aspects: Calculation of Binding Affinity

Information on the binding affinity of a ligand and on equilibrium binding constants can be directly extrapolated by the relative intensities of the peaks corresponding to different species in the *m*/*z* spectrum. In general, the results can be expressed on a semiquantitative level as the amount of complexed GQ that results from the relative intensities of complexed and free GQ, as the amount of uncomplexed GQ by monitoring the signal decrease of free nucleic acid in comparison to that of an internal standard, or by calculating the concentration of the bound ligand from relative peak intensities.^101−103^

First of all, peak intensities can be used to calculate macroscopic equilibrium binding constants. In this context, *K*_d_ is defined as [DNA][ligand]/[1:1 complex] and takes into account the total amount of the 1:1 complex, not considering ligand binding site(s).^85^ It must be assumed that both free and bound nucleic acid fractions have the same instrumental response upon ionization, which means that peak intensities reflect their relative concentrations in solution. This property is influenced by the ionization efficiency and several instrumental parameters. Generally, complexed and uncomplexed GQ species respond similarly to ESI, but summing the contributions of ion adduct peaks for each stoichiometry is required. It must be considered that species with similar *m*/*z* values transmit similarly, and species with the same charge state are detected with a similar efficiency. Thus, a quantitative investigation should be ideally carried out by considering the same charge state, while comparing assemblies of very different sizes should be avoided.^78,104^ Moreover, the separate determination of binding constants for each charge state, followed by an average calculation, is recommended.^28^ In their review, Rosu et al. detailed the equations that allow the calculation of the relative concentration of free nucleic acid and that of each complex, even with different stoichiometries, from peak areas. As precisely described by the authors, the total concentration of the bound ligand (and of the single complexes), as well as that of the free ligand at the equilibrium, can be retrieved from these data.^28^ Association constants (*K*_a_) with values from 10^3^ M^–1^ to 10^8^ M^–1^ can generally be determined by ESI-MS.^95,105^ Errors in the determination of binding constants may be due to complex disruption upon ionization or transit into the spectrometer (underestimation) or the fragmentation of the unbound nucleic acid (overestimation).^28^

ESI-MS provides the total mass of the complex, and the observed phenomenon corresponds to the macroscopic equilibrium binding constant without direct implications on the nature of the binding site and the binding mechanism. Nevertheless, information on microscopic binding constants in the presence of a limited number of binding sites can be indirectly deduced. As anticipated, compounds interacting with the GQ via π-stacking generally have two binding sites on the nucleic acid, corresponding to the external tetrads. Briefly, it must be assumed that the measured amount of the 1:1 complex includes the contributions of all the complexes containing one ligand independent from the position of the binding site. Considerations on the measured amount (*i.e.*, the peak area) of the 2:1 complex, as discussed more in detail by Rosu et al. in their review, give insights into the possible positive or negative cooperation, or independency, between the two binding sites.^28,100^

Since relative intensities (I) in a mass spectrum are assumed to be proportional to the concentrations of the species in the analyzed solution, the tendency of a compounds to form a complex with the nucleic acid, or in general with a target macromolecule, is also expressed in terms of the binding affinity (BA). This semiquantitative parameter can be directly calculated from the *m*/*z* spectrum using the following formula: BA = (Σ*I*_GQ bound_/(Σ*I*_GQ unbound_ + Σ*I*_GQ bound_)) × 100, where *I* is the relative intensity.^29,106^

In a briefer but similar fashion, results of ESI-MS screenings toward DNA can be also expressed as the concentration of the bound ligand per nucleic acid molecule. This can be calculated using the formula reported as follows: [bound ligand] = *C*_0_ × (*I*_(1:1)_ + 2*I*_(2:1)_ + 3*I*_(3:1)_)/(*I*_GQ unbound_ + *I*_GQ bound_). This calculation takes into account initial nucleic acid concentration (*C*_0_) as well as the formation of complexes with different stoichiometries.^31^

As previously anticipated, ESI-MS is also useful in the visual determination of the relative affinity of a ligand for different nucleic acid sequences. In a typical “selectivity screening” setup, the tested compound is incubated with a GQ, a dsDNA, and a single-strand sequence at the same time.^89,107^ Based on the graphical comparison of the amount of the bound ligand to the amount of different nucleotides, ESI-MS analysis promptly provides information on selectivity, assuming that the resolution is sufficient and complex peaks do not overlap. On the other hand, and using a similar approach, competition experiments using mixtures of several ligands can be performed.^100,108^

In light of its “instantaneous” nature, ESI-MS efficiently photographs the condition in a solution at a certain time point. Thus, ESI-MS can be used to study events showing sufficiently slow kinetics (minutes to hours), such as strand hybridization and formation or interconversion between different complexes.^51,109,110^

### Binding Mode

The first aspect that should be considered is that, since ESI-MS provides a single signal for every species detected to differ by mass, the stoichiometry of complexes can be directly deduced following the analysis. Several aspects of the stoichiometry can be investigated, including the number of nucleic acid strands, the number of complexed cations, and the number of molecules of bound ligands.^28^

**Figure 3:**
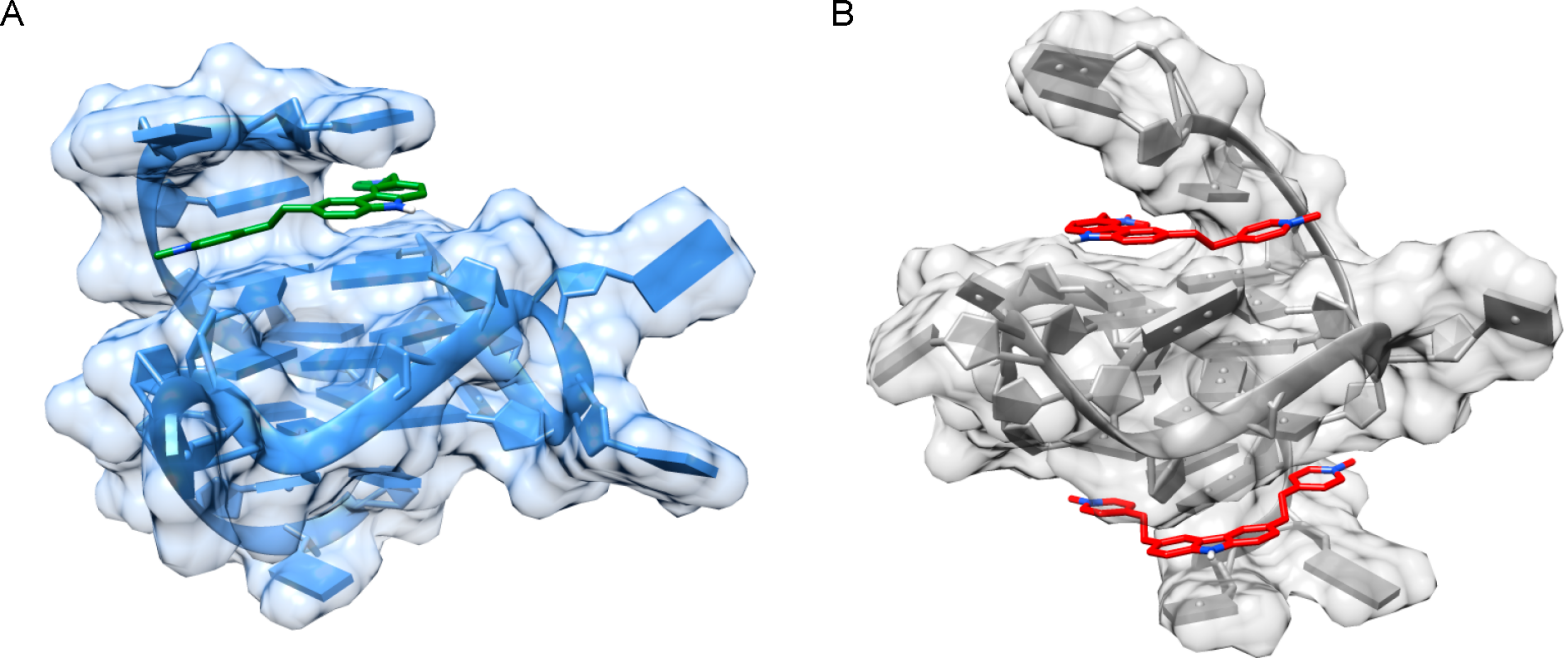
Carbazole derivative BMVC forms (A) 1:1 (PDB ID 6JJ0) and (B) 2:1 (PDB ID 6O2L) complexes with the c-MYC GQ.^111^

Mass information does not provide insights about the binding mode or the interaction site of a ligand to a GQ sequence *per se*. Nevertheless, some relevant clues can be retrieved from the *m*/*z* spectrum. As anticipated, ESI-MS is particularly convenient when it comes to stoichiometry characterization. The binding cooperativity and even low-abundant species with peculiar stoichiometries can be easily detected. As an example, a binding stoichiometry limited to a 2:1 ratio preliminarily suggests that the interaction occurs through external stacking. Technically, the binding stoichiometry could also be investigated by conventional spectroscopic studies through data fitting, but mass spectrometry provides this information directly. Ligand–target stoichiometry is a relevant aspect in the field of GQ binders, and the formation of both 1:1 and 2:1 complexes by the same ligand was previously demonstrated. An example is given by the structures determined by NMR spectroscopy of the complexes generated by the fluorescent carbazole derivative BMVC with the c-MYC GQ, where the binding occurs via stacking (Figure 3).^111^

On the other hand, the count of cation adducts can also provide additional information on the interaction, as ligand intercalation promotes the displacement of one of the cations stabilizing the GQ assembly. This event can be unambiguously observed by ESI-MS. It must be also considered that, in some other cases and depending on structure, the ligand interaction may promote the entrapment of an additional cation.^112^

**Figure 4:**
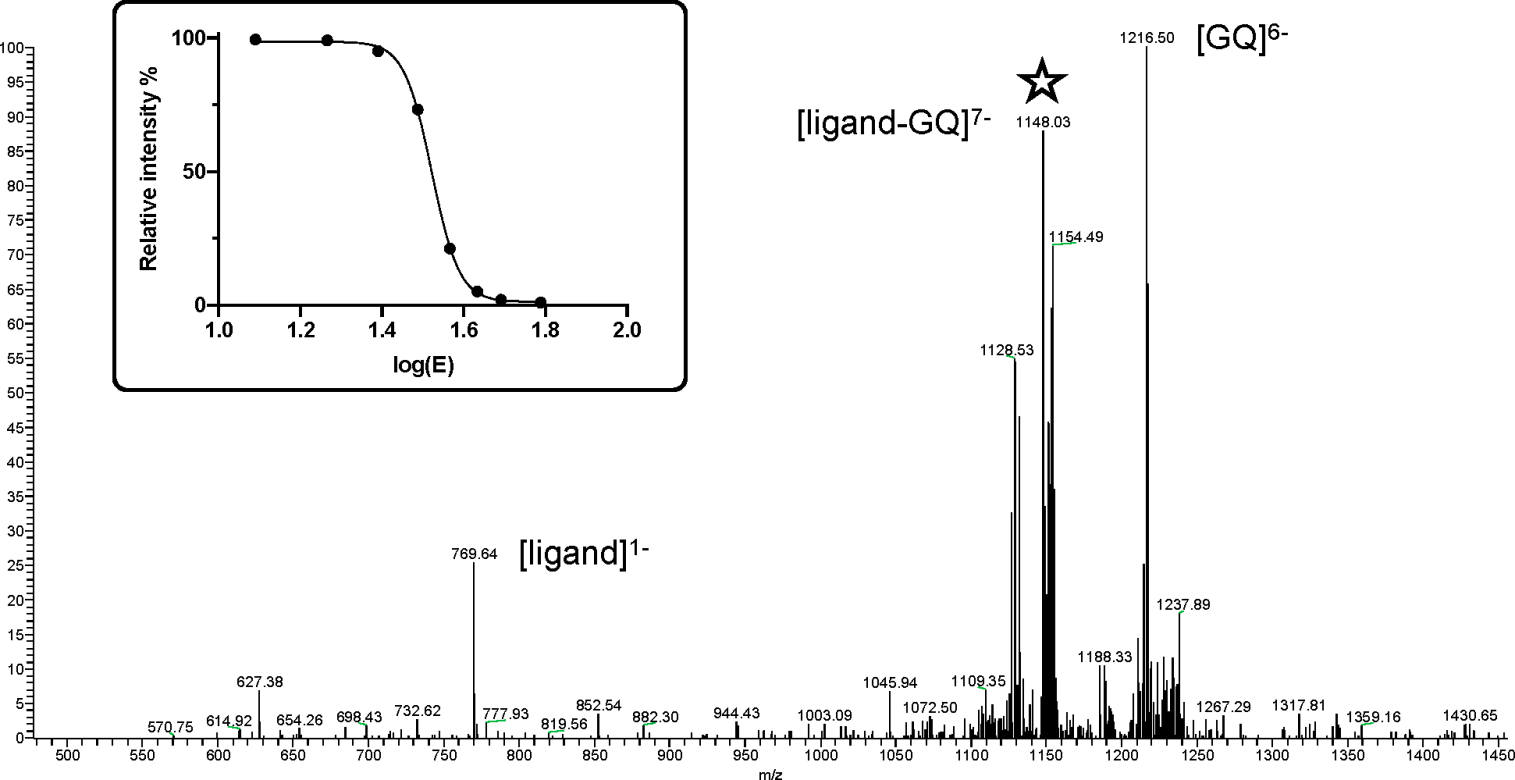
Example of the dissociation study carried out by our group. A ligand–GQ complex (5′-AGGGTTAGGGTTAGGGTTAGGGT-3′ human telomeric sequence), marked with a star in the spectrum, undergoes fragmentation by loss of the ligand. In the inset, the plot of the relative intensity of the complex against the collision energy (eV) is depicted.

In tandem mass spectrometry (MS/MS), an ion of a certain *m*/*z* is isolated and fragmented under opportune conditions using a gas that promotes dissociation by colliding with the species. This is due to the conversion of part of the relative kinetic energy into the vibrational energy of the ion (internal energy) that, when reaching a critical point, induces the fragmentation of the ion itself.^28^ Xu et al. investigated the collision-induced dissociation (CID) pattern for ligand–GQ complexes, highlighting that different fragmentation patterns correspond to different binding motifs. Interestingly, it was reported that complex fragmentation by the loss of the small molecule is associated with stacking interactions (Figure 4).^42,50^ Regardless, it must be taken into account that MS/MS experiments may induce fragmentation pathways and involve energies tha may not reflect native conformations. Moreover, such a pattern may also be influenced by the charge state and the gas phase basicity of the ligand.^60^

An additional complication is introduced by the fact that certain ligands can induce conformational switching in polymorphic sequences upon binding. In addition to this, it has been reported that different ligands preferentially induce different topologies on the same sequence. It must be borne in mind that the cation stoichiometry provides information on the number of stable quartets in the GQ. In high-resolution spectra, the ion adduct distribution of a GQ can be considered a distinguishing feature of a certain arrangement. If a ligand, when binding with a certain affinity and stoichiometry to the GQ, does not alter this distribution, the overall GQ structure should have been retained upon interaction. Regardless, it has been observed that ligands belonging to different chemical classes can indeed induce conformational changes.^82^ On the other hand, it must be pointed out that a direct correlation between the thermal stability and conformational switches cannot be extrapolated. In fact, a more complex network of equilibria is involved even if the interconversion proceeds through unfolding and refolding.^82,113^

Another strategy for determining the ligand binding site involves the use of covalent chemical probes.^114^ MS/MS, in this case, can also be performed on the labeled nucleic acid without the need to retain the native arrangement. Doria et al. reported the development of an oxirane derivativethat generated a stable adduct with the GQ. The selective alkylation of the loop adenines was detected, thus identifying the binding site.^115^

Ion mobility mass spectrometry (IM-MS) is another technique that allows one to gain structural information. Ions are pulsed in a chamber filled with a gas (helium). The time that is necessary for an ion of a certain *m*/*z* to travel to the mobility module is proportional to its “collision cross section”, which gives information on the overall size and stability of the complex. Most importantly, ions with multiple conformations (*i.e.*, interconverting topologies) require longer transition times.^5^ The reader is invited to refer to specific contributions in the field of ion mobility for a more detailed discussion of the technique.^28,116,117^ Concerning 3D arrangements and chirality, another cutting-edge technology consists of the combination of the MS-based separation of DNA strands and their characterization by CD. This techniques expands the possibilities of MS when applied to topology determination, which is also in the field of GQs.^118^

### Stability of the Ligand–GQ Complex: Dissociation Studies and Thermostabilization

Some assumptions must be made before discussing the application of MS/MS experiments to the study of ligand–GQ interactions. First, as anticipated, it must be considered that MS/MS experiments are performed on ligand–GQ complexes that are charged species isolated in a vacuum. Consequently, a correlation between the activation energy and the dissociation kinetic may not be trivial. Moreover, the fragmentation time scale changes depending on the adopted instrument. Thus, fragmentation spectra should be compared only when recorded on the same instrumental setup.^28^ Given the considerations reported in the previous paragraphs, the only pathway that can provide direct information on the energetic terms of GQ–ligand interactions is the loss of the neutral molecule from the negatively charged nucleic acid.^31,86^

Once that these preliminary assumptions have been considered, CID experiments can be used to investigate the relative gas-phase kinetic stability of complexes. Basing on the relative intensities of the adduct and the dissociation products in the *m*/*z* spectra recorded at increasing collision energies, the *E*_COM_^50%^ of the complex, which is expressed in electron volts, can be calculated. This value, which is elsewhere also defined as CE_50_, represents the center-of-mass collision energy needed to promote the dissociation of the complex to its relative half-intensity. The proceeding reaction, which is measured by MS/MS, consists in the dissociation of the ligand from the complex as follows: [ligand + GQ]^*z*−^·[GQ]^*z*−^ + ligand.^31^ Again, the value of *E*_COM_^50%^ can be directly calculated by using the relative intensities of such complexes and fragmentation products by plotting dissociation curves and using the following formula: *E*_COM_ = *I*_complex_/(*I*_complex_ + *I*_dissociation products_) (Figure 4).^31,119^ This parameter is of great relevance when evaluating small molecules as GQ binders, as GQ stabilization is one of the required properties for triggering biological effects.^31^

The issue of complex stability can be also approached by means of ESI-MS from another perspective. In fact, the thermostabilization of a GQ in response to ligand recognition can be also evaluated.^29,120^ Transition curves and *T*_50_ values for the dissociation of GQ structures can be calculated by acquiring spectra at increasing capillary temperatures. In a typical experimental setup, temperature can be increased from 60 to 400 °C, and decrease of the complex signal intensity can be measured to extrapolate *T*_50_.^88,121^

## Computational Studies

In the past two decades, the use of computer-aided drug design revolutionized the approach to the identification of small molecules targeting macromolecular assemblies and, more generally, the process of drug discovery. Virtual screening (VS) procedures allow the evaluation of thousands of compounds using receptor-based (also known as structure-based) and ligand-based techniques.^122^ On the other hand, modeling nucleic acids, and noncanonical arrangements in particular, can be challenging due to structural polymorphism, the presence of stabilizing metal ions, and sequence flexibility. Nevertheless, new methodologies and prediction algorithms emerged and have been optimized.^13,60,123,124^

Structure-based studies take advantage of the results from X-ray crystallography and NMR studies, and such 3D templates are used to screen large libraries of compounds.^125,126^ In general, structure-based VS aids the medicinal chemist in the first steps of the identification and rational optimization of GQ ligands.^60,127^

On the other hand, ligand-based methodologies use specific 2D or 3D queries to screen databases and highlight the best-matching compounds; molecules can be searched in light of their structure similarity or basedon more sophisticated 3D pharmacophore models.^128^ In general, a pharmacophore model can be generated starting from the analysis of the chemical structures and structural features of a set of known compounds with predetermined inhibitory activities or binding properties. The resulting 3D model is then used as a query to screen a library of compounds.^129^ In the past, this approach has been pursued to screen a library of nearly 10000 molecules using a pharmacophore model generated from 1,4-disubstiuted anthraquinone derivatives to identify novel potential GQ binders.^34^ In the field of ligand-based studies, it must be also reported that some molecular descriptors were found to correlate with binding properties. In particular, the analysis of conformational properties and the solvent-accessible surface area of known GQ ligands demonstrated that such physicochemical properties can be used for the prediction of the binding activity of π-stacking ligands in combination with the calculation of Boltzmann-averaged solvent-accessible surface area (BASASA).^130^

Thus, the study of the interaction between GQ arrangements and their putative ligands nowadays can be greatly enhanced by different computational methods. In the context of structure-based studies, molecular docking and molecular dynamics (MD) represent the most commonly used strategies to investigate the event of binding between a receptor, which can be either a protein or a nucleic acid, and a ligand by molecular mechanics (MM) methods. Moreover, particular force fields (ffs) that are constituted by functional forms and parameter sets regulate the energies that arise from the interactions involved.

Molecular docking applies a “search and score” method. The search algorithm explores all the possible positions and orientations of a screened ligand for its binding to a receptor that is generally considered as a rigid body. Moreover, the algorithm also explores all the possible conformations of the ligand by atom–atom bonds and dihedrals rotation. This search algorithm can be either geometric matching, incremental construction, Monte Carlo, genetic, ff-based, or a combination of these. For every generated docked pose, a binding energy value (−kcal/mol) is calculated through a scoring function that can be ff-based, empirical, knowledge-based, or a combination of these.^122^ An increased level of accuracy can be achieved by exploiting what is called the flexible ligand-flexible receptor approach. This can be pursued with different strategies, and one of them is ensemble docking. This technique relies on the simultaneous usage of multiple receptor structures, mainly obtained from different crystals of the same receptor or as frames of a MD simulation.^131^

MD can be described as a ff application for a system that is allowed to evolve over time from the starting conditions in a particular ensemble of variables, which can be either microcanonical ensemble (NVE), canonical (NVT), or isothermal–isobaric (NPT). The system, which can be constituted only by the receptor, by the ligand, or by the complex, is placed in an explicit solvent box. Depending on the time scale considered in the simulation, several different phenomena can be studied. In particular, side chain fluctuations, molecular tumbling, and helical folds can be observed in the nanosecond to microsecond time frame. Moreover, with simulations longer than 1 μs and up to the millisecond range and above, events such as protein folding can be detected.^132^ Important measurements can be derived from MD simulations, the most important of which is the root-mean-square deviation (RMSD) that can be used to assess the overall stability of the system. Moreover, root-mean-square fluctuations (RMSF) and the radius of gyration (rg) can be measured. In conclusion, it is important to cite the MM/PBSA and MM/GBSA methods for the free energy calculations, both of which are applicable to molecular docking or MD calculations.^133−137^ Besides MM-derived potential energies, these methods also take into account the free energy variations involved due to the solvation events occurring in consequence of binding in the presence of an implicit water model, which can be either Poisson–Boltzmann in the case of MM/PBSA or generalized Born and surface area continuum solvation in the case of MM/GBSA.^138^ On the basis of these fundamentals, a focus on the applications of computational methods in the particular field of the interaction of ligands with nucleic acids will be reported in the following.

### Molecular Docking

Molecular docking is a powerful tool for the computational investigation of binding modes of either a molecule or a set of molecules, which can be either newly synthesized or examined in the context of drug repurposing. When compared to MD, docking surely has the advantage of requiring relatively low computational resources, which allows the batch screening of entire ligand data sets by VS methods to be performed. Many large chemical libraries are currently available, such as MayBridge (http://www.maybridge.com.), AnalytiCon (https://ac-discovery.com/screening-libraries/), ZINC (http://zinc.docking.org/), ChemDiv (http://www.chemdiv.com/services-menu/screening-libraries/), SPECS (http://www.specs.net), Mcule (https://mcule.com/database/), eMolecules (https://www.emolecules.com/), PubChem (https://pubchem.ncbi.nlm.nih.gov/), Life Chemicals (http://www.lifechemicals.com/), and ChemBridge (https://www.chembridge.com/screening_libraries/). Different molecular docking software suitable for use with nucleic acid receptors have also been proposed, even if most of them were originally developed for protein targets. A brief description of their functions together with some applications retrieved from the literature in the context of the study of GQs are presented in the following.

AutoDock is one of the most widely used software programs and it is composed of two main distributions, namely AutoDock4 (AD4) and AutoDock Vina (Vina), which were respectively released in 2009 and 2010.^139,140^ The biggest difference between them consists of the scoring function that is semiempirical AMBER ff-based for AD4 and fully empirical for Vina. In addition to that, the latter has been demonstrated to be sensitively faster than AD4 by two orders of magnitude.^140^ Both AD4 and Vina can be used by command line and can also be implemented in graphical user interface (GUI) software like AutoDockTools (ADT), PyRx, and Raccoon, where these last two also allow VS to be run in batch. With a similar setup, Alcaro et al. identified a psoralene derivative as a new GQ ligand by screening 2.7 million molecules from the ZINC database. In particular, the molecules were subjected to ensemble docking by considering the four most representative GQ arrangements, namely the parallel and antiparallel arrangements and two mixed-type GQs with both parallel and antiparallel features.^141^ Cosconati et al. performed a structure-based VS in tandem with NMR experiments, finding six new GQ groove-binding compounds. The docking was performed with AD4, screening 6000 compounds from the Life Chemicals database and using the [d(TGGGGT)]_4_ GQ structure (PDB ID 1S45) as a receptor. By applying a binding energy and a cluster size filter cutoff and discarding the poses not showing peculiar interactions, 30 compounds were highlighted. Then, NMR titration experiments allowed the elimination of eight false positives to find six actual GQ groove binders. These preliminary results were also confirmed by authors with ITC experiments, suggesting good agreement between the computational and experimental data.^126,142^ Ranjan et al. reported the binding of aminosugar–intercalator conjugates, which are represented by derivatives of compounds from *Oxytricha nova*, with an antiparallel GQ. In this work, four conjugates between neomycin and common intercalators with different surface areas were studied. The BQQ–neomycin conjugate displayed the best binding to this DNA GQ structure, with an association constant (*K*_a_) of 1.01 ± 0.03 × 10^7^ M^–1^ that was nearly 100-fold higher than the binding of neomycin to the same GQ. The molecular modeling part of the study, performed with Vina, revealed for all the conjugates that neomycin was positioned in the wide groove with a linker extending the intercalating moieties more toward the thymine loop regions. Moreover, stacking interactions were observed only for the smallest polycyclic ring, the anthraquinone intercalator.^143^

Glide (grid-based ligand docking with energetics) is the docking engine of the Schrödinger suite. It relies on an exhaustive search algorithm where an initial rough positioning and scoring is followed by torsionally flexible energy optimization with the OPLS ff. The very best candidates are further refined via a Monte Carlo sampling, followed by a last selection of the best docked pose using a model energy function that combines empirical and ff-based terms.^144^ Glide offers three different precision settings intended to be used for different docking approaches from structural studies to VS. Moreover, specific VS tools are present in the Schrodinger suite, such as XGlide and the Virtual Screening Workflow. A structure-based VS on 31 000 natural compounds was realized by Artese et al. with this setup.^145^ The cited study comprehended the development of single pharmacophore hypotheses on already deposited crystals of ligand–telomeric GQ complexes; then, after the protocol validation, a VS was conducted with the Glide HTVS docking protocol using specific decoy sets. This allowed 12 final hits to be obtained for which the chemical scaffolds were already associated with GQ binding properties and antiproliferative effects. Kar et al. based their VS on a two-step Glide docking that consisted of a preliminary standard precision (SP) screening followed by an extra precision (XP) redocking of the top-scoring molecules.^146^ In particular, the NMR structure of the human telomeric GQ TAGGG(TTAGGG)_3_ (PDB ID 2lD8) was used. The placement of two potassium ions between the three quartets was followed by the identification of potential binding sites with SiteMap, and the sites were considered in the VS of the 14 000 molecules from the Maybridge database.^147^ Two selective GQ ligands were highlighted by this screening, and their performances were confirmed by fluorescence titrations. The ability of indenoisoquinoline topoisomerase inhibitors to bind and stabilize the GQ formed by the MYC promoter, thus downregulating MYC expression, was also studied in another contribution. In more detail, the docking protocol consisted of a Glide SP precision docking of a 7-azaindenoisoquinoline molecule to a previously resolved 2:1 complex. This approach produced binding poses that resembled those, previously obtained for quindoline in the NMR structure of the 2:1 quindoline–GQ complex, where a flanking DNA base from the 5′- or 3′-flanking segment was recruited to form ligand–base plane stacking over the external tetrads.^148^

DOCK relies on a ff-based scoring function and a rigid receptor-flexible ligand geometric matching (GM) algorithm. The latter uses a sampling algorithm called anchor-and-grow that is able of “building” the ligands into the active site of the receptor. In particular, the largest rigid scaffold of the ligand, namely the anchor, is identified, placed, and oriented in the binding site, then the flexible portions of the ligand are systematically added to the anchor to build the whole molecule. Starting from DOCK 6, an AMBER-based MD engine was implemented to account for receptor flexibility, allowing for rank ordering by energetic ensembles in the docking calculations. Wang et al. identified three new c-MYC GQ-stabilizing ligands using a combined approach that consisted of filtering 560 000 compounds from ChemDiv and SPECS libraries using a pharmacophore search and then performing docking with DOCK (ver. 5.4) to the TMPyP4-bound region of the NMR-derived c-MYC GQ (PDB ID 2A5R). The grid-based flexible docking results were rescored by GB/SA scoring. Three final compounds, characterized by different chemical scaffolds, showed a selective PCR-arresting effect, the inhibition of c-MYC transcription, and a decrease of promoter activity by binding to GQ in the promoter region without conformational changes of parallel-stranded GQ.^149^

ICM is the docking software developed by Molsoft. Its scoring function is based on an all-atom vacuum ff (ECEPP/3) with appended terms to account for the solvation free energy and the entropic contribution. The search algorithm consists of a biased probability Monte Carlo (BPMC) search algorithm. ICM was used by Lee et al. to screen the 20 000 molecules of the AnalytiCon Discovery GmbH library using a telomeric GQ (PDB code 1KF1) as the receptor.^150^ The five best-scoring compounds were tested in a polymerase stop assay, identifying the natural product fonsecin B as a stabilizing ligand for c-MYC GQ. The obtained docking pose revealed that the relatively flat scaffold is stacked on the GQ guanine quartet at the 3′ terminus; in this model, the phenolic and carbonyl oxygen atoms are situated close to the central potassium ion, possibly producing favorable electrostatic interactions.

### Molecular Dynamics (MD) Simulations

As discussed previously, MD simulations can be described as the application of a force field over time to a system of atoms that can belong to proteins, nucleic acids, or ligands. The most used ffs are called “additive” and consider charges as fixed and centered on atoms. On the other hand, “polarizable” ff add polarizable dipoles to atoms so that the charge description depends on the environment. The ff selection is crucial for MD simulations of DNA and particularly GQs. In fact, only few of them can accurately simulate these structures. Nevertheless, almost every ff can be easily implemented in different MD software. Among them, the most popular and widely adopted are GROMACS, AMBER, and DESMOND (https://www.gromacs.org/;^151^https://ambermd.org,^152^ and https://www.schrodinger.com(153)). The CHARMM27 all-atom additive ff for nucleic acids^154^ and with its evolution, the CHARMM36 all-atom additive ff for nucleic acids,^155^ are the specific CHARMM ffs for the investigation of these macromolecules and have been widely tested in the simulation of B-DNA, even if their efficacy with GQ has not been fully demonstrated. Fadrna et al. tested CHARMM27 for simulating two telomeric GQs, namely, the antiparallel d(G_4_T_4_G_4_)_2_ dimeric quadruplex with diagonal loops (PDB ID 1JRN) and the parallel-stranded human telomeric monomolecular quadruplex d[AGGG(TTAGGG)_3_] with three propeller loops (PDB ID 1KF1).^156^ In all cases, the ff mostly failed and produced a substantial instability. CHARMM36 was used as itself or in combination with the Drude polarizable ff to simulate different GQ structures, such as c-KIT1, c-KIT2, c-KIT, and BCL-2 promoters. In all cases, the polarizable version of the CHARMM36 ff demonstrated a superior reliability when reproducing the experimental structures. The simulations performed with CHARMM36, instead, suffered from inadequate ion interactions and instability, and the expulsion of one of the central ion was also observed.^149,157,158^

The AMBER parmbsc0 ff is a modification of parm99 that puts the emphasis on the correct representation of the α- or γ-concerted rotation in nucleic acids. As the authors reported, the ff was derived by fitting the models to high-level quantum mechanical data, which were verified by a comparison with high-level quantum mechanical calculations and a comparison between simulations and experimental data. Moreover, the validation study included long MD simulations and a large variety of nucleic acid structures.^159^ In 2015, the same group released a new version called parmbsc1, which includes the modifications present in parmbsc0 and additional improvements to the sugar pucker, the χ-glycosidic torsion, and the ε- and ζ-dihedrals.^160^

**Figure 5:**
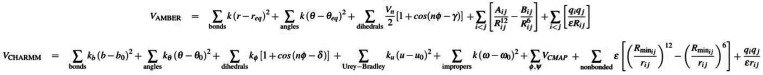
Functional forms for potential energy for AMBER and CHARMM. For both, the first sum regards covalent bonds, while the last one regards the Lennard-Jones and charge–charge interactions. The image was adapted from the Amber20 manual.

Another important ff for DNA simulation is OL15, which is based on parm99/bsco with additional modifications on the χ-, ε- or ζ-, and β-dihedrals of the sugar–phosphate backbone. Concerning the parametrization of a possibly present ligand that is complexed with DNA, this is generally accomplished using generic force fields compatible with the ones used for the receptors, namely, the CHARMM General Force Field (CGenFF) for the CHARMM ff and GAFF/GAFF2 for the AMBER ff (Figure 5). Using the OL15 and GAFF2 ffs for GQ and the ligand, respectively, Macchireddy et al. proved the binding mode of BRACO 19 to GQ using free-ligand MD simulations.^161^ The most stable binding mode was identified as end-stacking, and among the three screened GQ topologies (parallel, antiparallel, and hybrid) the MM-GBSA binding energy analysis suggested that the interaction with the parallel scaffold was the most energetically favorable for BRACO-19. Most importantly, the binding mode obtained using the apo GQ form (PDB ID 1KF1) was consistent with the structure of the complex of BRACO19 with an equivalent GQ structure retrieved by X-ray diffraction (PDB ID 3CE5).

A primary issue in MD simulations of GQ is the parametrization of the ions present in the GQ central channel. The choice of the Lennard-Jones parameter can play a central role in the stability of the structures and the retention of the ions. Havrila et al. tested different DNA and RNA GQs (PDB IDs 1KF1, 31BK, 1K8P, 143D, 2KF8, 2KM3, 2HY9, 2JPZ, 2MBJ, and 3QXR) with OL15 and a RNA-specific ff.^162^ The Joung–Cheatham (JC) SPC/E K^+^ parameters performed well for the majority of the simulated systems.^163^ The choice of the water model is also important and should be done by taking the other ffs used for the receptor and the ions into account; concerning GQ simulations, the most used ones are the three-site models SPC/E and TIP3P, which are often used in combination with JC ions parameters.^163^ Nevertheless, the four-site TIP4Pew demonstrated the ability to perform extremely well with JC parameters.^164^

## Conclusion and Perspectives: Combining ESI-MS and Computational Studies

The existence of a marked affinity and possible cooperation between ESI-MS studies and molecular modeling performed on small molecules targeting nucleic acids were envisaged previously, principally based on the consideration that both experiments are technically performed in a vacuum.^31^ Nevertheless, as discussed above, simulating native conditions and solvent influence is a primary aim of more sophisticated modern methods. More generally, docking studies have the potential to assist the researcher in interpreting the results from ESI-MS concerning the molecular recognition pattern. In fact, besides π-stacking, electrostatic interactions, which have been predicted by molecular modeling to play a primary role in the interaction with nucleic acids, can also be efficiently probed by ESI-MS.^28^ In a previous contribution, Rosu et al. investigated the possible correlation between the binding and selectivity of a set of compounds toward dsDNA and noncanonical nucleic acid sequences, which were retrieved from ESI-MS experiments, with the results of molecular modeling studies. In particular, docking energetics were calculated in the vacuum or gas phase; thus, the authors compared the results of such screening with MS/MS experiments and particularly with *E*_COM_^50%^ values.^31^

Taking the step from here, and starting from the results of previous docking and ESI-MS experiments carried out by our research group, we are currently setting up a preliminary study of the correlation between the calculated binding energy (Δ*G*) and *E*_COM_^50%^ for a pool of chemically diverse in-house GQ–ligands.^42−44,100^ This correlation, along with the hypothetical connection between the BA and the docking score, is being investigated on an increasing number of compounds. This would allow us to begin the design and implementation of a library of molecules to be used as a “training set” to tune the *in silico* discovery workflow, such as in a virtuous cycle. The subsequent step would be the application of this optimized computational prescreening algorithm to reduce the workload of ESI-MS experiments and further focus the efforts in the laboratory.

With the aim of pursuing the further optimization of computational techniques, induced fit protocols could be exploited or multiple GQ conformations could be generated by MD, either in solvent or in a vacuum, and then used in an ensemble docking approach to overcome the rigidity of the receptors. For what concerns the MD simulation of the GQ in a vacuum, D’Atri et al. proposed a more advanced model for simulating MS conditions.^5^ The GQ structure was modified, lowering the number of negative charges by the localized charge (LC) method. Then, short simulations were conducted with the parmbsc0 ff to obtain four snapshots, which were used as starting structures for four independent MD runs. In more detail, water molecules and external counterions were removed. No radial cutoff was set for Coulomb and van der Waals interactions, and the PME algorithm was not applied. This allowed them to obtain reliable models for the calculation of theoretical collision cross sections; in a vacuum, the G-core structure was maintained, and terminal thymine flipping was observed. Thus, the use of optimized docking and MD protocols in the VS workflow could improve the quality of the observed correlation, thus allowing the use of the resulting algorithm in a predictive fashion in the future.

In conclusion, novel experimental tools and methodologies are needed to better understand the involvement of GQs and the ligand–GQ interaction in diseases, with a particular focus on the molecular aspects of recognition. Moreover, more efficient and reliable screening tools are required to speed up the identification of potential hits in order to proceed faster with lead development. In this context, it must be considered that different techniques provide different and often complementary information on structure recognition and binding. ESI-MS can be intended as a rapid screening technique that has a low sample consumption and for which automation can be easily implemented. Stoichiometry, sensitivity, and accuracy are among the features that make ESI-MS a valuable tool for probing noncovalent interactions. Moreover, it allows some of the limitations connected to the use of more traditional techniques applied to the study of GQ structures to be overcome, such as difficulties related to sample preparation and large nucleic acid consumption. Proceeding in parallel with computational studies allows the valuable further refinement of the experimental data. Docking and MD data help with the interpretation of dissociation patterns, ion mobility data, and generally information on the interaction motif retrieved from ESI-MS studies. On the other hand, correlations such as the one highlighted between *E*_COM_^50%^ and Δ*G* could pave the way for building a hybrid screening workflow, where VS is used to screen a wider library of compounds and ESI-MS is applied to a set of best-scoring compounds. Regardless, it must be stressed that finely tuned and sophisticated simulation data, such as those retrieved from the ensemble and multiparameter docking of MD studies, are needed to lay the basis for a mixed virtual–experimental ligand discovery setup.

From the point of view of the medicinal chemist, the combination of ESI-MS and multilevel *in silico* investigations would result in a highly efficient hybrid high-throughput setup, providing fast and accurate feedback on the quantitative and structural aspects of ligand–GQ interactions.

## Abbreviations

AD4: AutoDock4
ADMET: adsorption, distribution, metabolism, excretion, and toxicity
ADT: AutoDockTools
BA: binding affinity
BASASA: Boltzmann-averaged solvent-accessible surface area
BPMC: biased-probability Monte Carlo
C_0_: initial nucleic acid concentration
CD: circular dichroism
CGenFF: CHARMM General Force Field
CID: collision-induced dissociation
DNA: deoxyribonucleic acid
dsDNA: double-stranded DNA
ESI-MS: electrospray ionization mass spectrometry
ff: force field
Glide: grid-based ligand docking with energetics
GM: geometric matching
GQ: G-quadruplex
GUI: graphical user interface
HIV: human immunodeficiency virus
*I*: relative intensity
IM-MS: ion mobility mass spectrometry
ITC: isothermal titration calorimetry
JC: Joung–Cheatham
*K*_a_: association constant
LC: localized charge
MALDI: matrix-assisted laser desorption ionization
MD: molecular dynamics
MM: molecular mechanics
MM/GBSA: molecular mechanics energies combined with the generalized Born and surface area continuum solvation
MM/PBSA: molecular mechanics energies combined with Poisson–Boltzmann and surface area continuum solvation
MS/MS: tandem mass spectrometry
NMR: nuclear magnetic resonance
NPT: isothermal–isobaric conditions
NVE: microcanonical ensemble conditions
NVT: canonical conditions
OPLS: optimized potentials for liquid simulations
PDB: Protein Data Bank
rg: radius of gyration
RMSD: root mean square deviation
RMSF: root mean square fluctuations
RNA: ribonucleic acid
SARS-CoV-2: severe acute respiratory syndrome-coronavirus-2
SP: standard precision
SPR: surface plasmon resonance
TRAP: telomere repeat amplification protocol
VS: virtual screening
XP: extra precision
